# Supplementary material for: Broadband Achromatic Metalens in the Visible Light Spectrum Based on Fresnel Zone Spatial Multiplexing
Source: Nanomaterials (Basel). 2022 Dec 3;12(23):4298. doi: 10.3390/nano12234298 (PMC9738994; doi:10.3390/nano12234298)
Supplement: Supplementary file 1 [file nanomaterials-12-04298-s001.zip › nanomaterials-2022381-supplementary.pdf]

Supplementary Information
Broadband Achromatic Metalens in the Visible Light Spectrum Based on Fresnel Zone Spatial Multiplexing

Ruixue Shi [1], Shuling Hu [1,*], Chuanqi Sun [1], Bin Wang [2,*] and Qingzhong Cai [1]

1 School of Instrumentation and Optoelectronics Engineering, Beihang University, Beijing 100191, China
2 Institute of Microelectronics of the Chinese Academy of Sciences, Beijing 100029, China
* Correspondence: hulxi@buaa.edu.cn (S.H.); wangbin3@ime.ac.cn (B.W.)

Note 1 The simulations of the metalens with different Fresnel zones

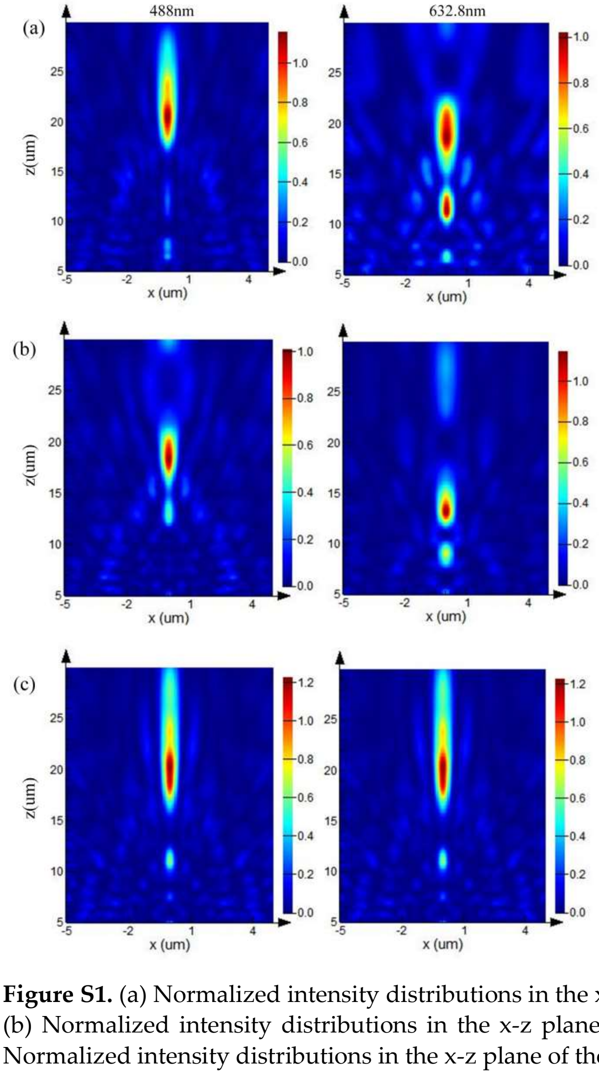

Figure S1. (a) Normalized intensity distributions in the x-z plane of the 1:1 Fresnel zones metalens; (b) Normalized intensity distributions in the x-z plane of the 1:1:1:1 Fresnel zones metalens; (c) Normalized intensity distributions in the x-z plane of the 3:2:1:1 Fresnel zones metalens.

Note 2 The diameters of the meta-atoms.

Table S1. Key parameters of meta-atoms used for the design of an achromatic metalens.

Zone	*Radius(nm)*
Zone I	86
	87
	88.5
	89
	90
	91.5
	99
	100
	103
	105
	109
	110
	112
	113
	168
	169
	170
	172
	174
	177
	178
	181
	183
	188
Zone II	78.5
	81
	87
	88
	89
	90
	92
	115
	116
	117
	119
	124
	125
	128
	129
	130
	132
	133
	136
Zone III	68
	86
	87
	88
	90

	98
	99
	100
	102
	105
	106
	107
	108
	111
	113
	168
	169
	170
	172
	184
Zone IV	88
	89
	90
	91
	115
	131
	134
	135
	140
